# Trends and patterns of broadband Internet access speed in a Nigerian university campus: A robust data exploration

**DOI:** 10.1016/j.dib.2019.103705

**Published:** 2019-02-02

**Authors:** Aderemi A. Atayero, Segun I. Popoola, Oluwaseun J. Adeyemi, David G. Afolayan, Matthew B. Akanle, Victor Adetola, Emmanuel Adetiba

**Affiliations:** a IoT-enabled Smart and Connected Communities (SmartCU) Research Cluster, Department of Electrical and Information Engineering, Covenant University, Ota, Nigeria; b Center for Systems and Information Services, Covenant University, Ota, Nigeria; c HRA, Institute for Systems Science, Durban University of Technology, Durban, South Africa

**Keywords:** Smart campus, Broadband internet access, Data bit rate, Mobile communication, Knowledge management

## Abstract

Efficient broadband Internet access is required for optimal productivity in smart campuses. Besides access to broadband Internet, delivery of high speed and good Quality of Service (QoS) are pivotal to achieving a sustainable development in the area of education. In this data article, trends and patterns of the speed of broadband Internet provided in a Nigerian private university campus are largely explored. Data transmission speed and data reception speed were monitored and recorded on daily basis at Covenant University, Nigeria for a period of twelve months (January–December, 2017). The continuous data collection and logging were performed at the Network Operating Center (NOC) of the university using SolarWinds Orion software. Descriptive statistics, correlation and regression analyses, Probability Density Functions (PDFs), Cumulative Distribution Functions (CDFs), Analysis of Variance (ANOVA) test, and multiple comparison post-hoc test are performed using MATLAB 2016a. Extensive statistical visualizations of the results obtained are presented in tables, graphs, and plots. Availability of these data will help network administrators to determine optimal network latency towards efficient deployment of high-speed broadband communication networks in smart campuses.

**Specifications table**

**Table:** 

Subject area	*Engineering*
More specific subject area	*Internet Engineering*
Type of data	*Tables, graphs, figures, and Microsoft Excel spreadsheet file*
How data was acquired	*The continuous data collection and logging were performed at the Network Operating Center (NOC) of the university using SolarWinds Orion software.*
Data format	*Raw, analyzed*
Experimental factors	*All statistical computations were performed using MATLAB 2016a*
Experimental features	*Various statistical visualizations such as boxplots, time series plots, frequency distributions, correlation and regression analyses, Probability Density Functions (PDFs), Cumulative Distribution Functions (CDFs), Analysis of Variance (ANOVA) test, and multiple post-hoc test performed on the dataset are presented. MATLAB 2016a software was used for the statistical computations.*
Data source location	*The dataset on broadband Internet access speed presented in this article were collected at Covenant University, Ota, Nigeria (Latitude 6.6718°N, Longitude 3.1581°E)*
Data accessibility	*Data is with this data article as supplementary material to aid reproducible research. This data is hosted in Mendeley data repository:*https://data.mendeley.com/datasets/c9kcbf4s6t/1
	doi:10.17632/c9kcbf4s6t.1
Related research article	*S. N. John, C. Ndujuiba, R. Okonigene, and N. Kenechukwu, "Simulation and Monitoring of a University Network for Bandwidth Efficiency Utilization," in Proceedings of the International Conference on Modeling, Simulation and Visualization Methods (MSV), 2013, p. 1.*

**Value of the data**

• The data provided in this data article include both peak and off-peak periods and these are valuable to the development of prediction or forecasting models for broadband communication networks in a smart campus environment [1], [2].
• Robust data exploration presented in this data article will facilitate effective bandwidth distribution and allocation based on need, priority, and desired Quality of Service [3], [4], [5].
• Open access publication of these empirical data has an inherent ability to spur further evidence-based research on efficient bandwidth allocation and usage in computer networking [6], [7], [8].
• Availability of these data will help network administrators to determine optimal network latency towards efficient deployment of high-speed broadband communication networks in smart campuses [9], [10], [11].

## 1 Data

**Fig. 1:**
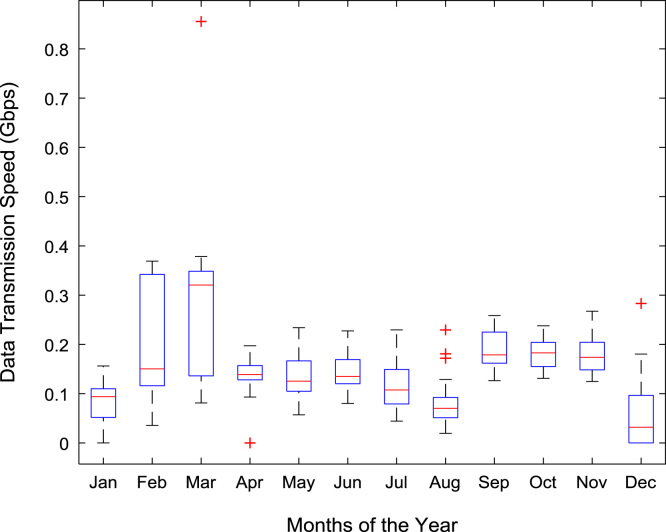
Boxplot representation of data transmission speed in Gbps.

**Fig. 2:**
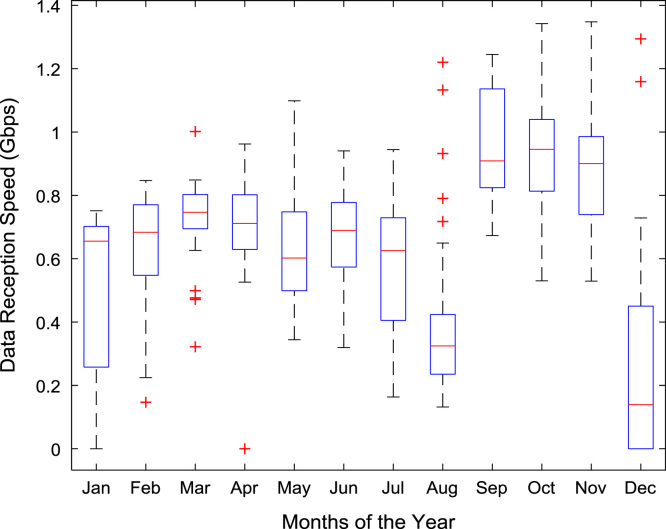
Boxplot representation of data reception speed in Gbps.

**Fig. 3:**
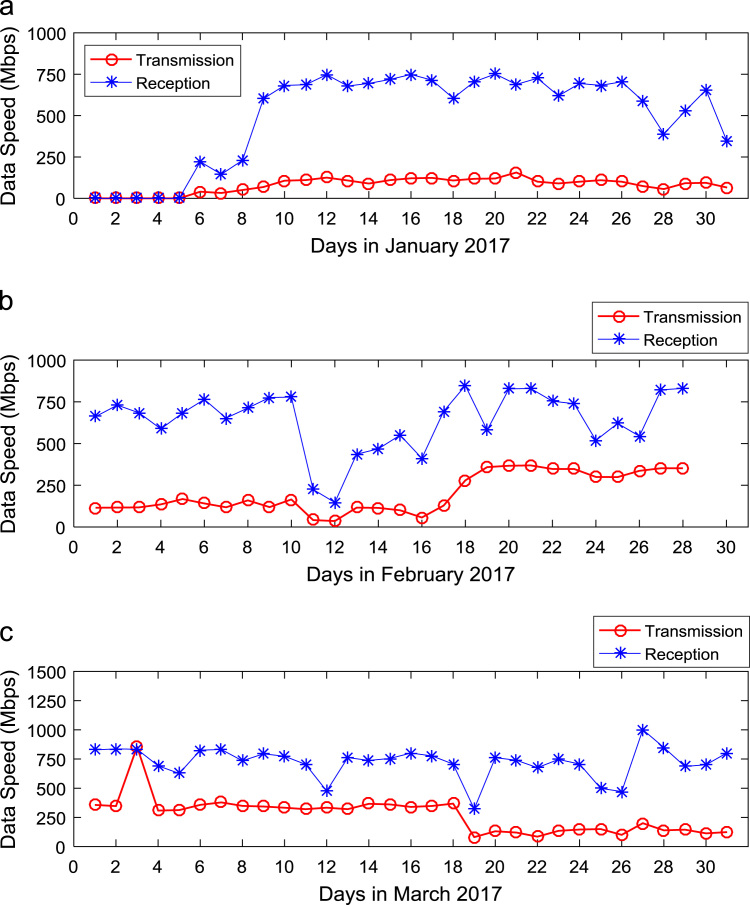
Trends of Internet speed in (a) January (b) February and (c) March 2017.

**Fig. 4:**
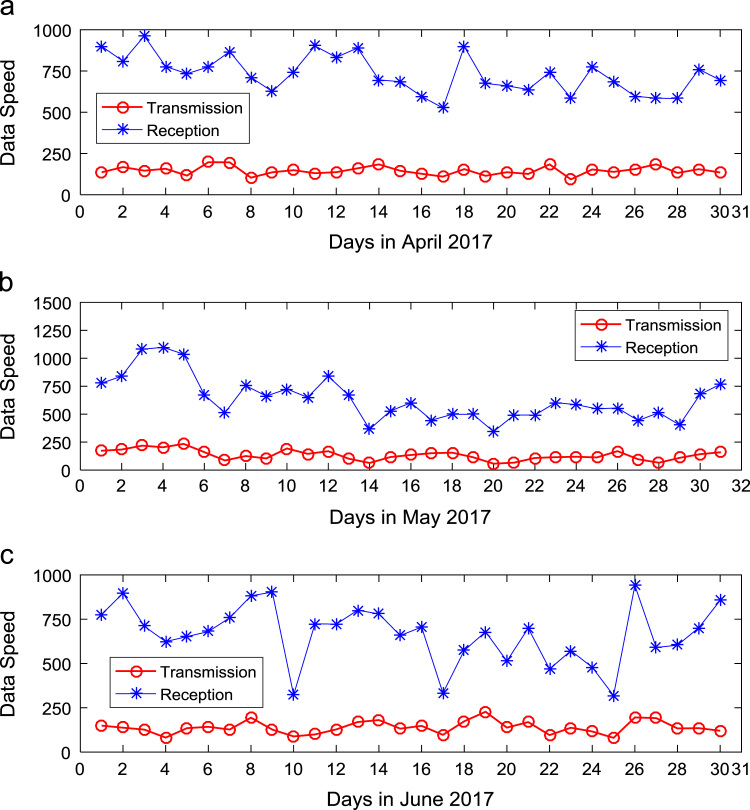
Trends of Internet speed in (a) April (b) May and (c) June 2017.

**Fig. 5:**
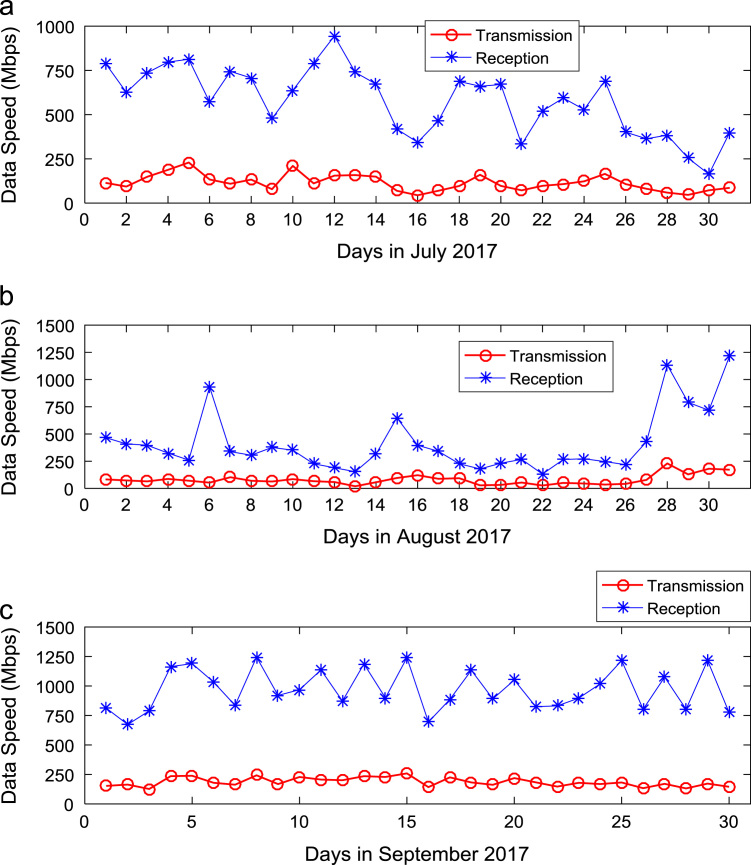
Trends of Internet speed in (a) July (b) August and (c) September 2017.

**Fig. 6:**
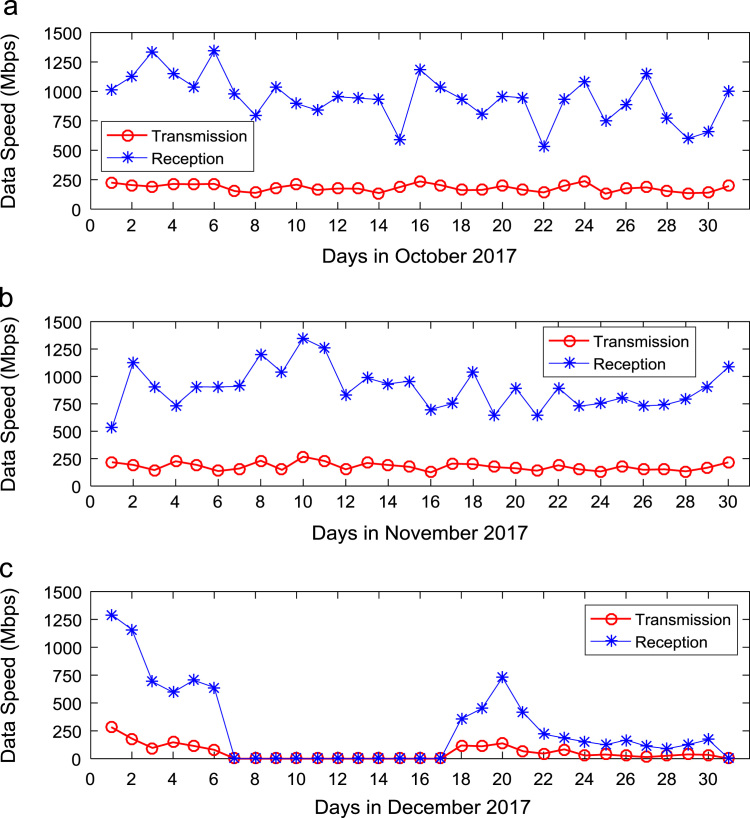
Trends of Internet speed in (a) October (b) November and (c) December 2017.

**Fig. 7:**
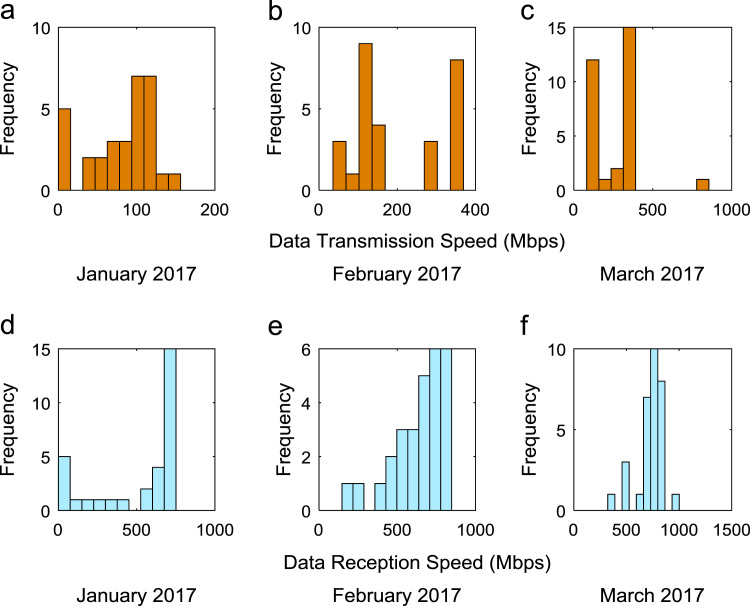
(a)–(f). Frequency distributions of Internet speed in the first quarter of 2017.

**Fig. 8:**
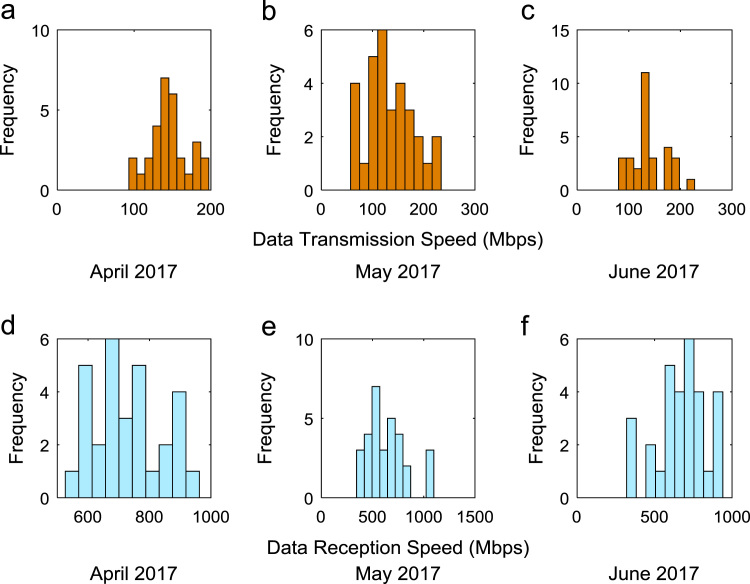
(a)–(f). Frequency distributions of Internet speed in the second quarter of 2017.

**Fig. 9:**
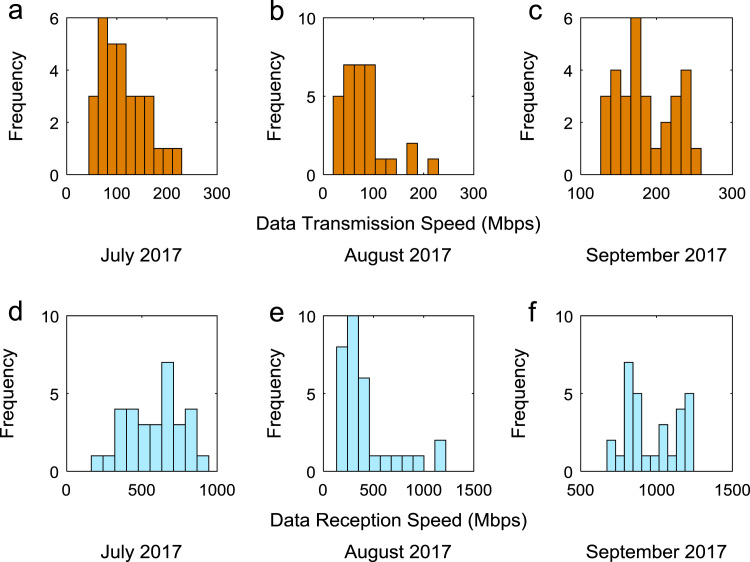
(a)–(f). Frequency distributions of Internet speed in the third quarter of 2017.

**Fig. 10:**
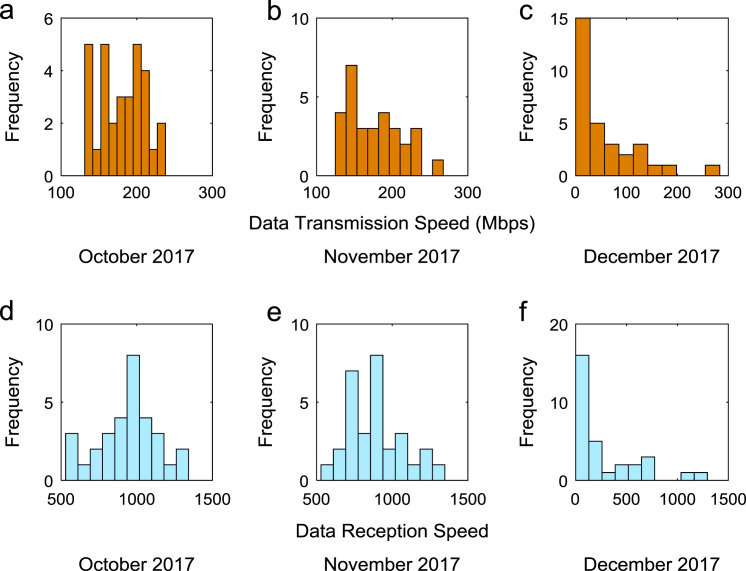
(a)–(f). Frequency distributions of Internet speed in the fourth quarter of 2017.

Quantitative data on broadband Internet access speed in Covenant University are presented in a reusable format. The data presented are further explored to reveal useful insights that are needed for productive decision making based on statistical parameters used in [13], [14], [15], [16], [17], [18]. Datasets on Internet transmission and reception speeds are extensively described by their statistical mean, median, mode, standard deviation, variance, kurtosis, Skewness, range, minimum, maximum, and sum as shown in Table 1 and Table 2 respectively. Fig. 1 and Fig. 2 show the quartiles, minimum, maximum, and outliers in the transmission data and the reception data using boxplots. Trends of broadband Internet access speed in the university were analyzed monthly and the resulting graphs for each quarter of the year 2017 are shown in Fig. 3, Fig. 4, Fig. 5, Fig. 6. Similarly, the frequency distributions of the data are shown in Fig. 7, Fig. 8, Fig. 9, Fig. 10.

**Table 1:** Descriptive statistics of data transmission speed in Gigabit per second (Gbps).

	*Jan*	*Feb*	*Mar*	*Apr*	*May*	*Jun*	*Jul*	*Aug*	*Sep*	*Oct*	*Nov*	*Dec*
Mean	0.08	0.20	0.27	0.14	0.13	0.14	0.12	0.08	0.19	0.18	0.18	0.06
Median	0.09	0.15	0.32	0.14	0.13	0.14	0.11	0.07	0.18	0.18	0.17	0.03
Mode	0.00	0.04	0.32	0.00	0.06	0.14	0.04	0.02	0.13	0.13	0.12	0.00
Standard Deviation	0.04	0.12	0.15	0.04	0.05	0.04	0.05	0.05	0.04	0.03	0.04	0.07
Variance	0.00	0.01	0.02	0.00	0.00	0.00	0.00	0.00	0.00	0.00	0.00	0.00
Kurtosis	2.37	1.48	7.60	8.12	2.44	2.78	2.85	5.20	1.92	1.98	2.49	5.19
Skewness	− 0.68	0.30	1.55	− 1.61	0.26	0.42	0.64	1.49	0.28	0.06	0.50	1.50
Range	0.16	0.33	0.77	0.20	0.18	0.15	0.19	0.21	0.13	0.11	0.14	0.28
Minimum	0.00	0.04	0.08	0.00	0.06	0.08	0.04	0.02	0.13	0.13	0.12	0.00
Maximum	0.16	0.37	0.86	0.20	0.23	0.23	0.23	0.23	0.26	0.24	0.27	0.28
Sum	2.47	5.66	8.37	4.36	4.15	4.18	3.57	2.46	5.57	5.59	5.35	1.67

**Table 2:** Descriptive statistics of data reception speed in Gigabit per second (Gbps).

	*Jan*	*Feb*	*Mar*	*Apr*	*May*	*Jun*	*Jul*	*Aug*	*Sep*	*Oct*	*Nov*	*Dec*
Mean	0.50	0.64	0.72	0.71	0.63	0.66	0.58	0.41	0.97	0.94	0.89	0.28
Median	0.66	0.68	0.75	0.71	0.60	0.69	0.63	0.32	0.91	0.95	0.90	0.14
Mode	0.00	0.15	0.32	0.00	0.34	0.32	0.16	0.13	0.67	0.53	0.53	0.00
Standard Deviation	0.28	0.18	0.13	0.17	0.20	0.17	0.19	0.28	0.18	0.20	0.19	0.36
Variance	0.08	0.03	0.02	0.03	0.04	0.03	0.04	0.08	0.03	0.04	0.04	0.13
Kurtosis	2.22	3.87	4.82	10.05	3.22	2.74	2.27	4.92	1.73	2.87	2.95	4.16
Skewness	− 0.93	− 1.14	− 1.13	-2.06	0.87	− 0.49	− 0.28	1.67	0.17	− 0.11	0.52	1.39
Range	0.75	0.70	0.68	0.96	0.75	0.62	0.78	1.09	0.57	0.81	0.82	1.29
Minimum	0.00	0.15	0.32	0.00	0.34	0.32	0.16	0.13	0.67	0.53	0.53	0.00
Maximum	0.75	0.85	1.00	0.96	1.10	0.94	0.94	1.22	1.25	1.34	1.35	1.29
Sum	15.55	17.88	22.46	21.92	19.68	19.93	17.93	12.76	29.07	29.22	26.68	8.38

**Fig. 11:**
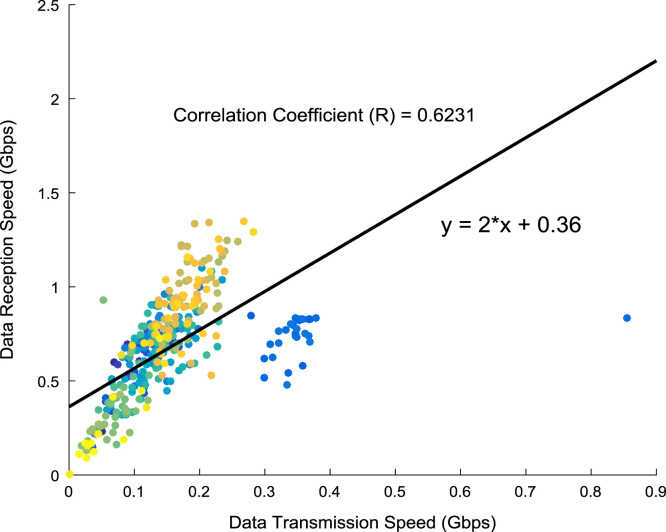
Scatter plot of broadband Internet access speed.

**Fig. 12:**
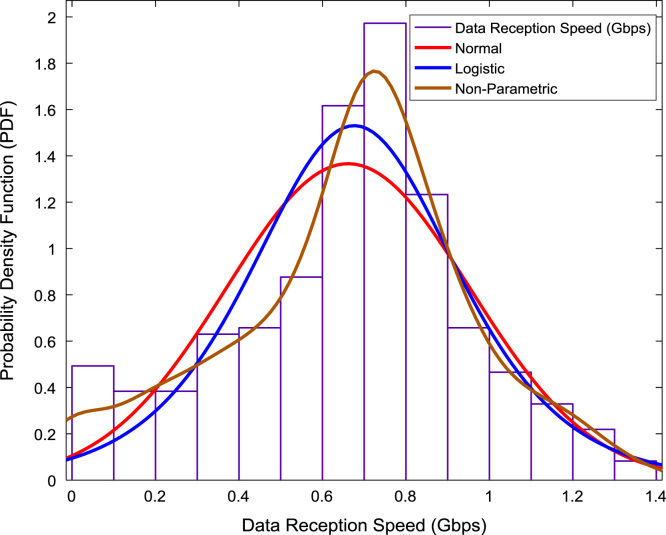
Probability distributions of data transmission speed.

**Fig. 13:**
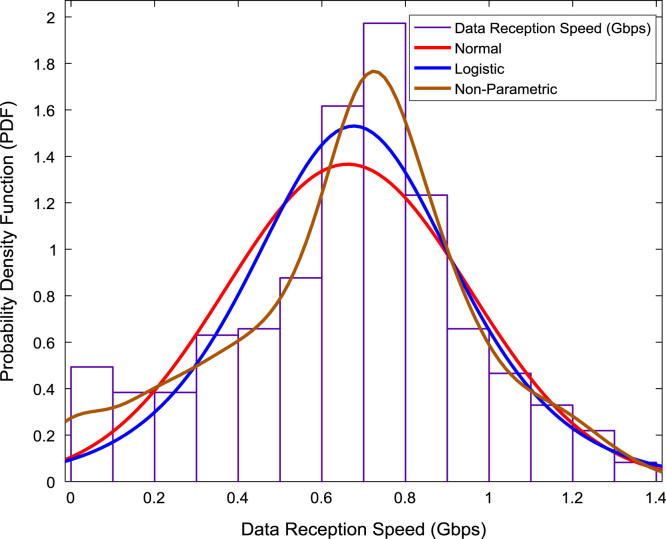
Probability distributions of data reception speed.

**Fig. 14:**
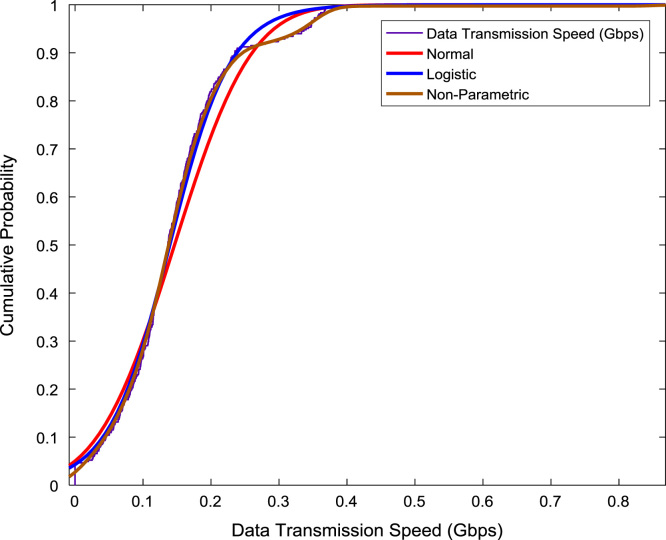
Cumulative probability distributions of data transmission speed.

**Fig. 15:**
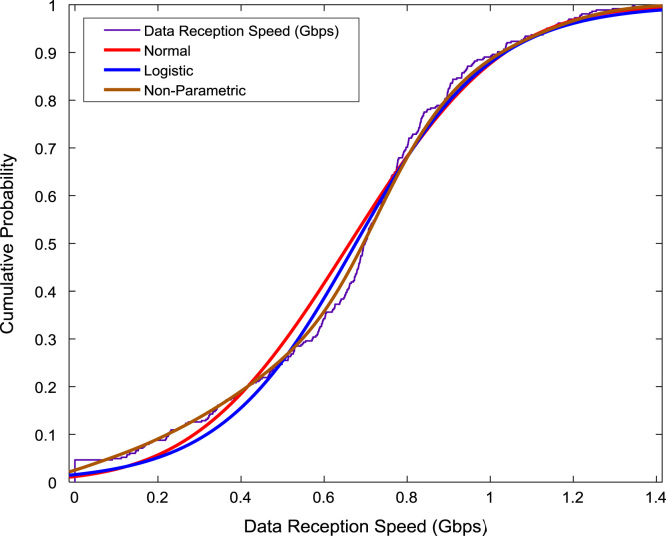
Cumulative probability distributions of data reception speed.

The scatter plot shown in Fig. 11 illustrates the relationship between the data transmission speed and the data reception speed that were monitored and logged daily for a period of twelve months. A regression line, linear regression equation, and regression coefficient are made available on the scatter plot. In addition, probability distributions of the transmission speed and the reception speed were computed and the results are presented in Fig. 12 and Fig. 13 respectively. In like manner, the cumulative densities of the datasets are shown in Fig. 14 and Fig. 15. The Distribution fitting parameters for data transmission speed and data reception speed are presented in Table 3 and Table 4 respectively. The estimates and standard errors of the two datasets are given in Table 5 and Table 6.

**Table 3:** Distribution fitting parameters for data transmission speed (Gbps).

	Normal	Logistic
Log Likelihood	365.399	394.714
Domain	− ∞ < y < ∞	− ∞ < y < ∞
Mean	0.1463	0.1394
Variance	0.0079	0.0066

**Table 4:** Distribution fitting parameters for data reception speed (Gbps).

	Normal	Logistic
Log Likelihood	− 68.1229	− 68.5258
Domain	− ∞ < y < ∞	− ∞ < y < ∞
Mean	0.6615	0.6763
Variance	0.0853	0.0878

**Table 5:** Estimates and standard errors of data transmission speed (Gbps).

	Normal		Logistic	
Parameter	Approx	Std Err	Approx	Std Err
µ	0.1463	0.0047	0.1394	0.0040
Σ	0.0890	0.0033	0.0449	0.0020

**Table 6:** Estimates and standard errors of data reception speed (Gbps).

	Normal		Logistic	
Parameter	Approx	Std Err	Approx	Std Err
µ	0.6615	0.0153	0.6763	0.0148
Σ	0.2920	0.0108	0.1633	0.0072

**Fig. 16:**
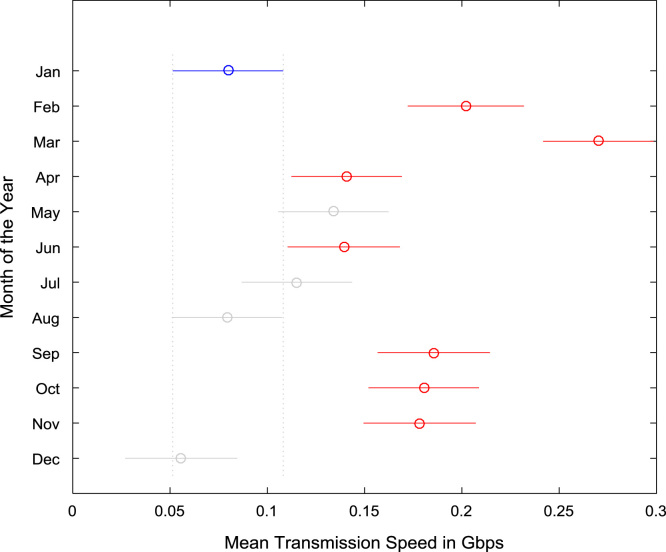
Graphical representation of monthly mean data transmission speed (Gbps).

**Fig. 17:**
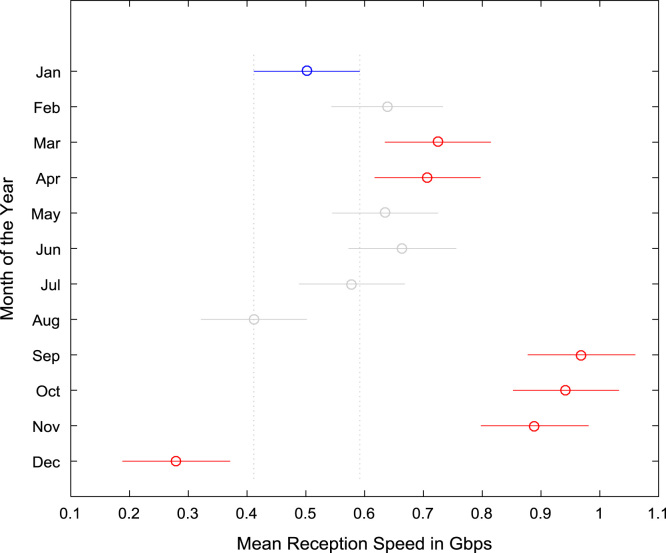
Graphical representation of monthly mean data reception speed (Gbps).

The datasets were tested for statistical difference across the months of the year based on Analysis of Variance (ANOVA) and multiple post-hoc comparison tests. The results of the ANOVA and multiple post-hoc comparison tests for data transmission speed are presented in Table 7 and Table 8 respectively. Similarly, the results of the ANOVA and multiple post-hoc comparison tests for data reception speed are presented in Table 9 and Table 10 respectively. Graphical representations of the results showing statistical difference in data transmission speed and data reception speed are shown in Fig. 16 and Fig. 17.

**Table 7:** ANOVA test results for data transmission speed (Gbps).

Source of Variation	Sum of Squares	Degree of Freedom	Mean Squares	F Statistic	Prob>F
Columns	1.2348	11	0.1123	24	9.41 ×10^–37^
Error	1.6511	353	0.0047		
Total	2.8859	364

**Table 8:** Multiple comparison post-hoc test results for data transmission speed (Gbps).

Groups Compared		Lower limits for 95% confidence intervals	Mean Difference	Upper limits for 95% confidence intervals	*p*-value
Jan	Feb	− 0.1805	− 0.1223	− 0.0640	0.0000
Jan	Mar	− 0.2471	− 0.1903	− 0.1336	0.0000
Jan	Apr	− 0.1177	− 0.0610	− 0.0042	0.0228
Jan	May	− 0.1109	− 0.0542	0.0026	0.0782
Jan	Jun	− 0.1167	− 0.0595	− 0.0022	0.0333
Jan	Jul	− 0.0922	− 0.0354	0.0214	0.6678
Jan	Aug	− 0.0563	0.0005	0.0573	1.0000
Jan	Sep	− 0.1630	− 0.1058	− 0.0485	0.0000
Jan	Oct	− 0.1574	− 0.1006	− 0.0438	0.0000
Jan	Nov	− 0.1557	− 0.0985	− 0.0412	0.0000
Jan	Dec	− 0.0332	0.0240	0.0813	0.9688
Feb	Mar	− 0.1264	− 0.0681	− 0.0098	0.0075
Feb	Apr	0.0030	0.0613	0.1196	0.0291
Feb	May	0.0098	0.0681	0.1264	0.0074
Feb	Jun	0.0040	0.0628	0.1215	0.0241
Feb	Jul	0.0286	0.0869	0.1451	0.0001
Feb	Aug	0.0645	0.1228	0.1810	0.0000
Feb	Sep	− 0.0422	0.0165	0.0752	0.9990
Feb	Oct	− 0.0366	0.0217	0.0799	0.9879
Feb	Nov	− 0.0350	0.0238	0.0825	0.9764
Feb	Dec	0.0876	0.1463	0.2050	0.0000
Mar	Apr	0.0726	0.1294	0.1861	0.0000
Mar	May	0.0794	0.1362	0.1930	0.0000
Mar	Jun	0.0736	0.1309	0.1881	0.0000
Mar	Jul	0.0982	0.1550	0.2117	0.0000
Mar	Aug	0.1341	0.1909	0.2476	0.0000
Mar	Sep	0.0273	0.0846	0.1418	0.0001
Mar	Oct	0.0330	0.0897	0.1465	0.0000
Mar	Nov	0.0346	0.0919	0.1491	0.0000
Mar	Dec	0.1571	0.2144	0.2716	0.0000
Apr	May	− 0.0500	0.0068	0.0636	1.0000
Apr	Jun	− 0.0558	0.0015	0.0587	1.0000
Apr	Jul	− 0.0312	0.0256	0.0823	0.9482
Apr	Aug	0.0047	0.0615	0.1182	0.0206
Apr	Sep	− 0.1020	− 0.0448	0.0124	0.3039
Apr	Oct	− 0.0964	− 0.0396	0.0171	0.4899
Apr	Nov	− 0.0948	− 0.0375	0.0197	0.5920
Apr	Dec	0.0278	0.0850	0.1422	0.0001
May	Jun	− 0.0626	− 0.0053	0.0519	1.0000
May	Jul	− 0.0380	0.0188	0.0755	0.9955
May	Aug	− 0.0021	0.0547	0.1114	0.0719
May	Sep	− 0.1089	− 0.0516	0.0056	0.1248
May	Oct	− 0.1032	− 0.0464	0.0103	0.2395
May	Nov	− 0.1016	− 0.0443	0.0129	0.3204
May	Dec	0.0210	0.0782	0.1354	0.0005
Jun	Jul	− 0.0331	0.0241	0.0813	0.9683
Jun	Aug	0.0027	0.0600	0.1172	0.0303
Jun	Sep	− 0.1040	− 0.0463	0.0114	0.2674
Jun	Oct	− 0.0984	− 0.0411	0.0161	0.4425
Jun	Nov	− 0.0967	− 0.0390	0.0187	0.5430
Jun	Dec	0.0258	0.0835	0.1412	0.0001
Jul	Aug	− 0.0209	0.0359	0.0927	0.6470
Jul	Sep	− 0.1276	− 0.0704	− 0.0131	0.0034
Jul	Oct	− 0.1220	− 0.0652	− 0.0084	0.0095
Jul	Nov	− 0.1203	− 0.0631	− 0.0059	0.0165
Jul	Dec	0.0022	0.0594	0.1167	0.0337
Aug	Sep	− 0.1635	− 0.1063	− 0.0490	0.0000
Aug	Oct	− 0.1579	− 0.1011	− 0.0443	0.0000
Aug	Nov	− 0.1562	− 0.0990	− 0.0418	0.0000
Aug	Dec	− 0.0337	0.0235	0.0808	0.9734
Sep	Oct	− 0.0521	0.0052	0.0624	1.0000
Sep	Nov	− 0.0504	0.0073	0.0650	1.0000
Sep	Dec	0.0721	0.1298	0.1875	0.0000
Oct	Nov	− 0.0551	0.0021	0.0594	1.0000
Oct	Dec	0.0674	0.1246	0.1819	0.0000
Nov	Dec	0.0648	0.1225	0.1802	0.0000

**Table 9:** ANOVA test results for data reception speed (Gbps).

Source of Variation	Sum of Squares	Degree of Freedom	Mean Squares	F Statistic	Prob>F
Columns	14.3882	11	1.3080	27.73	1.57 ×10^–41^
Error	16.6523	353	0.0472		
Total	31.0405	364

**Table 10:** Multiple comparison post-hoc test results for data reception speed (Gbps).

Groups Compared		Lower limits for 95% confidence intervals	Mean Difference	Upper limits for 95% confidence intervals	*p*-value
Jan	Feb	− 0.3220	− 0.1369	0.0481	0.3938
Jan	Mar	− 0.4033	− 0.2231	− 0.0428	0.0031
Jan	Apr	− 0.3858	− 0.2055	− 0.0253	0.0106
Jan	May	− 0.3136	− 0.1333	0.0470	0.3948
Jan	Jun	− 0.3444	− 0.1627	0.0191	0.1323
Jan	Jul	− 0.2570	− 0.0767	0.1036	0.9655
Jan	Aug	− 0.0903	0.0900	0.2703	0.8980
Jan	Sep	− 0.6492	− 0.4674	− 0.2856	0.0000
Jan	Oct	− 0.6212	− 0.4409	− 0.2606	0.0000
Jan	Nov	− 0.5696	− 0.3878	− 0.2060	0.0000
Jan	Dec	0.0403	0.2221	0.4038	0.0038
Feb	Mar	− 0.2712	− 0.0862	0.0989	0.9350
Feb	Apr	− 0.2537	− 0.0686	0.1164	0.9882
Feb	May	− 0.1814	0.0036	0.1887	1.0000
Feb	Jun	− 0.2123	− 0.0257	0.1608	1.0000
Feb	Jul	− 0.1249	0.0602	0.2452	0.9961
Feb	Aug	0.0418	0.2269	0.4119	0.0036
Feb	Sep	− 0.5170	− 0.3305	− 0.1440	0.0000
Feb	Oct	− 0.4891	− 0.3040	− 0.1190	0.0000
Feb	Nov	− 0.4374	− 0.2509	− 0.0644	0.0007
Feb	Dec	0.1725	0.3590	0.5455	0.0000
Mar	Apr	− 0.1628	0.0175	0.1978	1.0000
Mar	May	− 0.0905	0.0898	0.2700	0.8995
Mar	Jun	− 0.1214	0.0604	0.2422	0.9953
Mar	Jul	− 0.0340	0.1463	0.3266	0.2505
Mar	Aug	0.1327	0.3130	0.4933	0.0000
Mar	Sep	− 0.4262	− 0.2444	− 0.0626	0.0007
Mar	Oct	− 0.3982	− 0.2179	− 0.0376	0.0045
Mar	Nov	− 0.3465	− 0.1647	0.0171	0.1201
Mar	Dec	0.2633	0.4451	0.6269	0.0000
Apr	May	− 0.1080	0.0722	0.2525	0.9781
Apr	Jun	− 0.1389	0.0429	0.2247	0.9998
Apr	Jul	− 0.0515	0.1288	0.3091	0.4514
Apr	Aug	0.1152	0.2955	0.4758	0.0000
Apr	Sep	− 0.4437	− 0.2619	− 0.0801	0.0002
Apr	Oct	− 0.4157	− 0.2354	− 0.0551	0.0012
Apr	Nov	− 0.3640	− 0.1822	− 0.0005	0.0487
Apr	Dec	0.2458	0.4276	0.6094	0.0000
May	Jun	− 0.2111	− 0.0294	0.1524	1.0000
May	Jul	− 0.1237	0.0566	0.2369	0.9972
May	Aug	0.0430	0.2233	0.4036	0.0030
May	Sep	− 0.5159	− 0.3341	− 0.1524	0.0000
May	Oct	− 0.4879	− 0.3076	− 0.1274	0.0000
May	Nov	− 0.4363	− 0.2545	− 0.0727	0.0003
May	Dec	0.1736	0.3554	0.5371	0.0000
Jun	Jul	− 0.0959	0.0859	0.2677	0.9281
Jun	Aug	0.0708	0.2526	0.4344	0.0003
Jun	Sep	− 0.4880	− 0.3048	− 0.1215	0.0000
Jun	Oct	− 0.4601	− 0.2783	− 0.0965	0.0000
Jun	Nov	− 0.4084	− 0.2251	− 0.0419	0.0035
Jun	Dec	0.2014	0.3847	0.5680	0.0000
Jul	Aug	− 0.0136	0.1667	0.3470	0.1022
Jul	Sep	− 0.5725	− 0.3907	− 0.2089	0.0000
Jul	Oct	− 0.5445	− 0.3642	− 0.1839	0.0000
Jul	Nov	− 0.4928	− 0.3111	− 0.1293	0.0000
Jul	Dec	0.1170	0.2988	0.4806	0.0000
Aug	Sep	− 0.7392	− 0.5574	− 0.3756	0.0000
Aug	Oct	− 0.7112	− 0.5309	− 0.3506	0.0000
Aug	Nov	− 0.6595	− 0.4778	− 0.2960	0.0000
Aug	Dec	− 0.0497	0.1321	0.3139	0.4234
Sep	Oct	− 0.1553	0.0265	0.2083	1.0000
Sep	Nov	− 0.1036	0.0796	0.2629	0.9599
Sep	Dec	0.5062	0.6895	0.8728	0.0000
Oct	Nov	− 0.1286	0.0532	0.2349	0.9985
Oct	Dec	0.4812	0.6630	0.8448	0.0000
Nov	Dec	0.4266	0.6098	0.7931	0.0000

## 2 Experimental design, materials, and methods

A smart campus relies on robust and efficient broadband internet access for optimal functionality [12]. A case in point is Covenant University, Nigeria which currently has a subscription of seven Synchronous Transport Module level one (STM-1) from three Internet Service Providers (ISPs). For this massive investment to be justifiably utilized, precise knowledge of internet speed trend and pattern on both the uplink and downlink is essential. Besides access to broadband Internet, delivery of high speed and good Quality of Service (QoS) are pivotal to achieving a sustainable development in the area of education. In this data article, trends and patterns of the speed of broadband Internet provided in a Nigerian private university campus are largely explored. The data presented in this article will help in network planning towards guaranteeing desired QoS.

Covenant University, an ICT-driven private university located in Nigeria, is serviced with high-speed broadband Internet by three ISPs through fiber optic communication links. Two of the ISPs utilize STM-1 with an equivalent maximum Internet speed of 310 Megabit per second (Mbps) while the third ISP provides three STM-1 with an equivalent maximum Internet speed of 465 Mbps. All fiber optic communication links terminated at the Network Operating Center (NOC), which distributes available broadband Internet access to all academic, administrative, and residential buildings in the university campus. The data transmission speed and the data reception speed were monitored and recorded on daily basis for a period of twelve months (January – December, 2017). The continuous data collection and logging were performed with the use of SolarWinds Orion software. The network monitoring tool was installed on the bare metal server in the NOC to ensure sufficient computing resources. To facilitate easy data reuse for reproducible research, empirical data obtained from the experimental process were properly sorted and preprocessed using Microsoft Excel (MS-Excel) 2013 version.
